# Complete Mitochondrial Genome Sequence of the Magpie-Lark (Grallina cyanoleuca)

**DOI:** 10.1128/MRA.00342-21

**Published:** 2021-06-17

**Authors:** Subir Sarker, Babu Kanti Nath, Saranika Talukder, Shane R. Raidal

**Affiliations:** a Department of Physiology, Anatomy and Microbiology, School of Life Sciences, La Trobe University, Melbourne, Victoria, Australia; b School of Animal and Veterinary Sciences, Faculty of Science, Charles Sturt University, Wagga Wagga, New South Wales, Australia; c School of Agriculture and Food, Faculty of Veterinary and Agricultural Sciences, The University of Melbourne, Melbourne, Victoria, Australia; Vanderbilt University

## Abstract

Here, we report the complete mitochondrial genome sequence of an Australian passerine bird, magpie-lark (Grallina cyanoleuca). The circular genome has a size of 16,933 bp and contains 13 protein-coding genes, 22 tRNA genes, and 2 rRNA genes. This study provides a reference mitochondrial genome of magpie-lark for further molecular studies.

## ANNOUNCEMENT

The order Passeriformes (“perching birds”) is the largest order of birds and the dominant avian group on Earth today. With more than 140 families and some of 6,500 identified species, Passeriformes is one of the most diverse orders of terrestrial vertebrates (1). Magpies are members of the crow tribe, under the family Monarchidae, which is composed over of 100 passerine birds, including shrikebills, paradise flycatchers, and magpie-larks. The magpie-lark (Grallina cyanoleuca) is native to Australia, Timor, and southern New Guinea. Molecular studies on the magpie-lark (*G. cyanoleuca*) are limited, and only partial mitochondrial sequences of this species are available in the NCBI database (2, 3). The complete mitogenome sequence of *G. cyanoleuca* has not been characterized, and the taxonomic status and phylogenetic relationships of this species within the family Monarchidae remain unclear. Therefore, the present study aimed to sequence a mitogenome of *G. cyanoleuca* and determine its phylogenetic relationships.

A cutaneous tissue sample from a wild magpie-lark was collected at Canley Heights Veterinary Clinic, NSW, Australia (sample identifier [ID], 19-1000; global positioning system [GPS] latitude, 33°52′57.432′′S; longitude, 150°55′26.688′′E), and was deposited at Charles Sturt University. Animal sampling was performed following approved guidelines set by the Australian Code of Practice for the Care and Use of Animals for Scientific Purposes and approved by the Charles Sturt University Animal Ethics Committee (research authority permit 09/046). The genomic DNA (gDNA) was isolated using a ReliaPrep genomic DNA tissue miniprep system (Promega, USA) (4, 5). The library was prepared with 10 ng of total gDNA using the QIAseq FX DNA library kit (Qiagen) and sequenced using the Illumina NextSeq 500 platform, generating 150-bp paired-end reads (6–9). Sequencing data were analyzed following an established pipeline (10, 11) using Geneious (version 10.2.2; Biomatters, New Zealand) and CLC Genomics Workbench (version 9.5.4). Briefly, a total of 11.70 million raw reads were preprocessed to remove the Qiagen universal adapter, ambiguous base calls, and poor-quality reads (trim using quality score, limit 0.05; trim ambiguous nucleotides up to 15 using CLC Genomics Workbench), followed by mapping against an Escherichia coli bacterial genomic sequence (GenBank accession number U00096) to remove possible bacterial contamination. Trimmed and unmapped clean reads were used as input data for *de novo* assembly in CLC Genomics Workbench (version 9.5.4). This resulted in the generation of a 16,933-bp mitogenome obtained from *G. cyanoleuca*. A total of 9.10 million clean reads were mapped back to the mitogenome of *G. cyanoleuca* that resulted in an average coverage of 53.76×. Annotation of the assembled mitogenome of *G. cyanoleuca* was performed using default parameters under the genetic code of vertebrate mitochondria (transl_table 2) in Geneious (version 10.2.2). The final mitogenome of *G. cyanoleuca* was circularized using Geneious software under default parameters.

**FIG 1:**
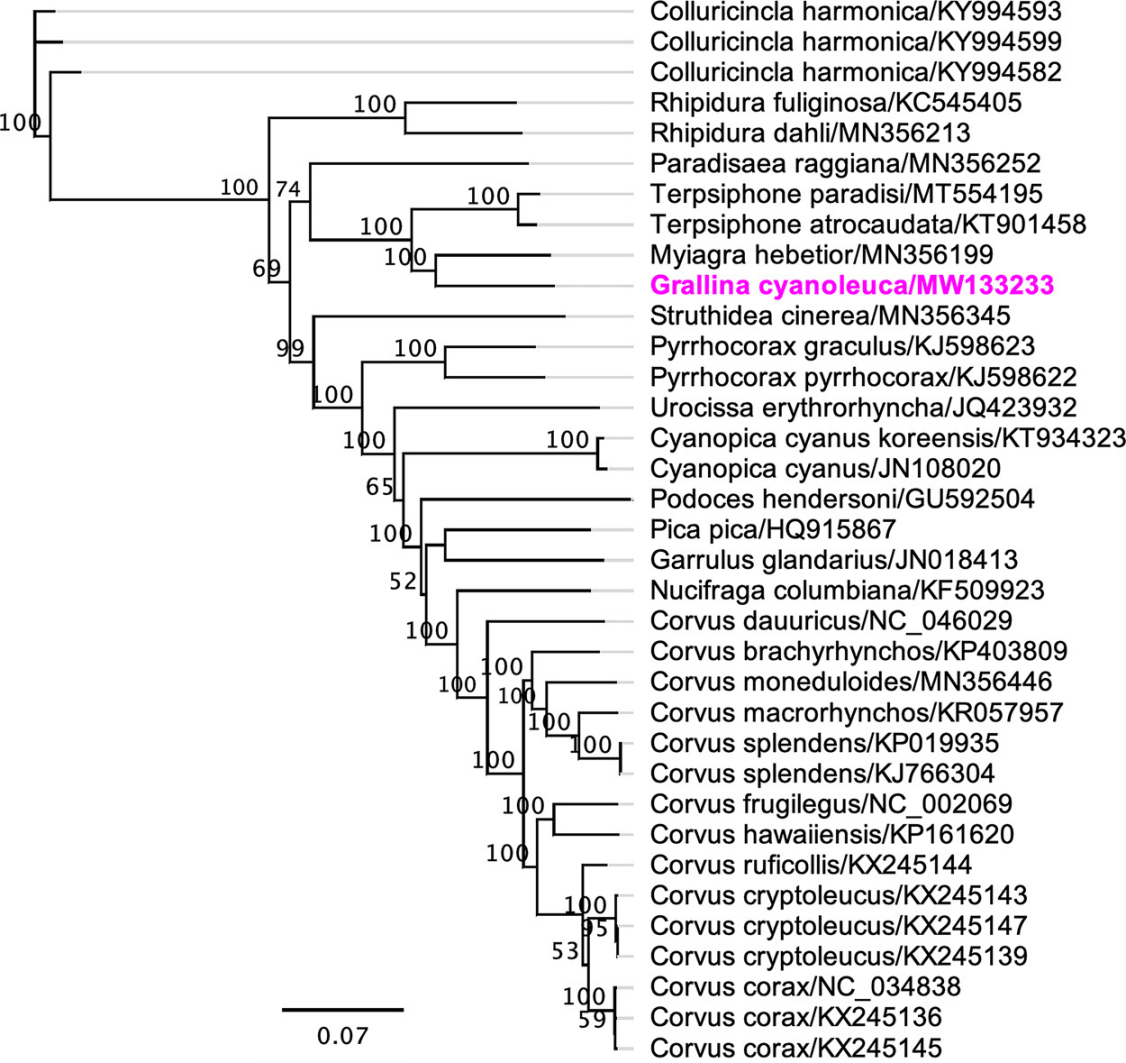
Maximum likelihood phylogenetic tree to infer host-phylogeny relationship using a mitochondrial genome sequenced from *G. cyanoleuca* along with other selected species under the order Passeriformes in the GenBank database. The nucleotide sequences were aligned with MAFFT (version 7.450) using G-INS-i (gap open penalty, 1.53; offset value, 0.123) algorithm implemented in Geneious (version 7.388) (14). A phylogenetic tree was performed under the GTR substitution model with 500-bootstrap support in Geneious (version 10.2.2). The complete mitochondrial genome of *G. cyanoleuca* is highlighted by bold font and magenta color. Labels at branch tips refer to species and GenBank accession numbers, separated by a slash.

The assembled complete mitogenome of *G. cyanoleuca* had a circular genome of 16,933 bp with a G+C content of 44.1%. Our annotation identified 1 control region (D-loop), 2 rRNA regions (12S and 16S), 22 tRNA genes, and 13 protein-coding genes (PCGs), and the gene content of the mitogenome of *G. cyanoleuca* was consistent with that of the other members of the order Passeriformes (12, 13). As highlighted in Fig. 1, the mitogenome sequence of *G. cyanoleuca* grouped into a strongly supported subclade with the mitogenome sequence from velvet flycatcher (Myiagra hebetior; GenBank accession number MN356199) (100% bootstrap support) and demonstrated a 90.36% pairwise nucleotide identity between them. We concluded that the complete mitogenome of *G. cyanoleuca* will be a useful database among the family Monarchidae for further studying the host-phylogenetic relationship.

### Data availability.

The complete mitochondrial genome sequence of *G. cyanoleuca* has been deposited in DDBJ/ENA/GenBank under the accession number MW133233. The version described in this paper is the first version, MW133233.1. Raw sequencing data from this study have been deposited in the NCBI Sequence Read Archive (SRA) under the accession number SRR14381441 (BioProject accession number PRJNA726748; BioSample accession number SAMN18960061).
